# Mandela’s generation: 99 years old and still thriving and surviving – this is the story of Mama Matlala

**DOI:** 10.3402/gha.v9.33899

**Published:** 2016-11-14

**Authors:** Jocelyn Anstey Watkins

**Affiliations:** Warwick Medical School, The University of Warwick, Coventry CV4 7AL, UK, MRC/Wits Rural Public Health and Transitions Research Unit (Agincourt), Acornhoek, Mpumalanga, South Africa

**Figure:**
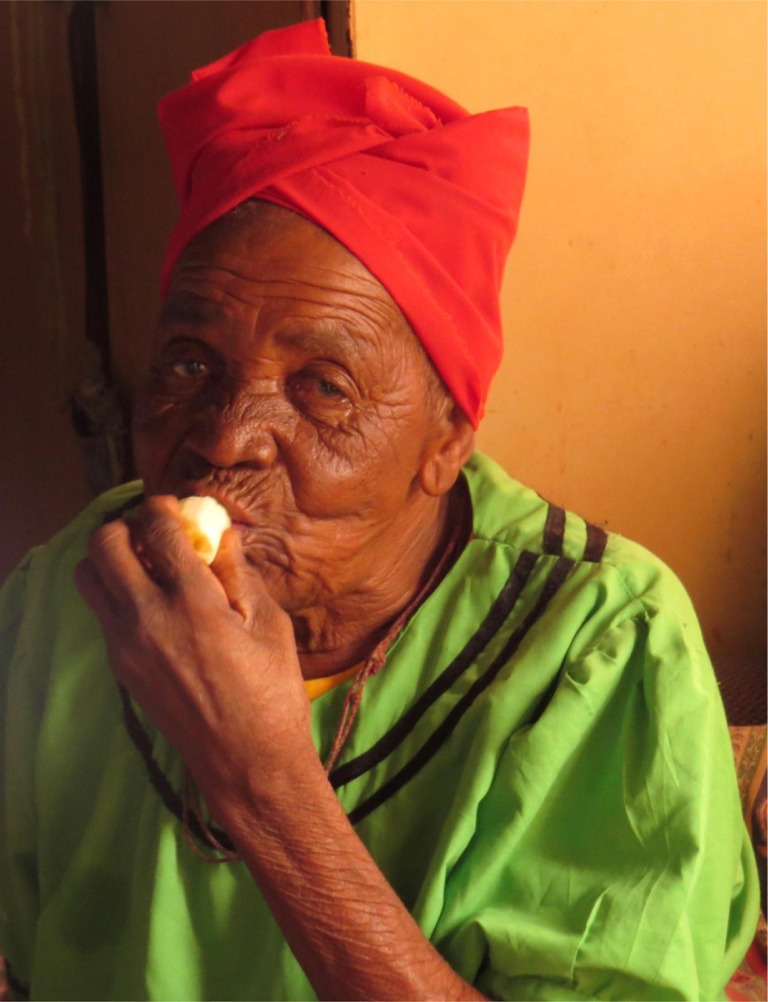


This photograph portrays my Mama Matlala, a 99-year-old Sotho women, a mother figure, who cared for me as a child. It was taken at her home in a village below a mountain, in the Limpopo Province, of South Africa, in February 2016.

This image captures her eating a soft banana from one of her trees, growing behind her hut. It is one of the few foods she can now eat, as she has no teeth left. Each day dresses in her traditional clothes and tends to her goats. She was sheltering inside, as it was a sweltering summer day, whilst her grandson collected water from the river for them to drink and cook with.

The image relates to many articles in the GHA as it epitomizes an older rural woman, who lacks access to healthcare despite it being free at the point of access. Mama can see only very faintly, as she has cataracts in both eyes, and her nearest primary healthcare clinic continually runs out of the medication to manage her hypertension. Despite this, she is still active and content.

Mama was the impetus for my own PhD research in rural South Africa on health system strengthening and the use of mobile health for patients with chronic disease. She represents a story of successful aging and perhaps more attention needs to be paid to older people like her in South Africa and beyond. Thriving in an environment like this is truly an act of survival and the spirit of human endurance. More research into the factors that contribute to Mama’s longevity should be accounted for, particularly surrounding diet and exercise so that the older generation can live long and healthy lives.

This is her life. This is her story.

*Jocelyn Anstey Watkins* Warwick Medical School The University of Warwick Coventry CV4 7AL, UK MRC/Wits Rural Public Health and Transitions Research Unit (Agincourt) Acornhoek, Mpumalanga, South Africa Email: Jotawatkins@gmail.com

